# Notes and Notices

**Published:** 1884-01

**Authors:** 


### NOTES AND NOTICES.

New Treatment of Cholera.—"Cholera" is prominently characterized among other symptoms by its vomiting and purging. A new allopathic plan of treating this dread disease is by emetics and purgatives. Verily the "world do move!"

On Bandaging the Infant.—Douglas Galton, President of the British Sanitary Institute, delivered a philippic against the baby-bandage. He condemned "those mischievous two yards of calico," which, wound round the middle of infants on their introduction into the world, constricted and hindered the expansion of that very region of the body where heart and lungs, stomach and liver, organs of no mean importance, were struggling for room to grow and do their work, and where natural mechanical conditions threw some difficulty in the way of full development.

A New Potentizer.—Dr. George F. Foote has lately invented a new potentizer—one that is guaranteed to produce none but genuine Hahnemannian dilutions. Dr. Foote's machine may be very accurate in its workings, but the water of Philadelphia is so thick and muddy that a sausage grinder would be necessary to potentize *it!*

The New Speaker.—Speaker Carlisle is a free-trader, so is the American Institute. It is currently reported that the Speaker would therefore endeavor to have Congress recognize Homœopathy if he could find any Homœopathy to recognize!

Query.—What potency is the "scent" which a dog will follow so cleverly? Could a microscopist *see* it?

A la Dante.—In order to prevent the I. H. A. from meeting in the same place as the Institute, let the latter inscribe over the door of its headquarters: "Let all who enter *here* leave Homœopathy behind."

An Important Omission.—Alphonse Karr was lately a guest at a dinner of some homœopathic physicians at Paris, when, after toasts had been honored to Hahnemann and to the great lights of the science now living, he was asked to propose a toast. "Gentlemen," he said, "you have drunk the health of many physicians, but there is one toast you have forgotten. Permit me to repair the omission. I drink to the health of your patients."—*Exchange.*

